# A Prospective Observational Study to Determine the Energy and Protein Adequacy in Children Aged Two to Five Years in the Urban Field Practice Area of Bangalore Medical College and Research Institute, Bengaluru, India

**DOI:** 10.7759/cureus.91367

**Published:** 2025-08-31

**Authors:** Rose Treesa Mathew, Jyothi Jadhav, Anu Priya Mathew C, Farsana Nathankodan, Vishnu Jayarajan, Selvi Thangaraj

**Affiliations:** 1 Community Medicine, Bangalore Medical College and Research Institute, Bengaluru, IND; 2 Community Medicine, Karuna Medical College, Palakkad, IND

**Keywords:** child food poverty, children, energy adequacy, food taboos, malnutrition, protein adequacy

## Abstract

Introduction: Diet plays a crucial role in a child's growth. Inadequate dietary intake in early childhood can lead to failure of children reaching their growth potential. The objective is to assess the energy and protein adequacy and to estimate child food poverty among children aged two to five years in the urban field practice area of Bangalore Medical College and Research Institute, Bengaluru, India.

Methodology: A prospective observational study was conducted among 110 children, selected using a simple random sampling method, and data were collected using a pretested, semistructured questionnaire. Malnutrition was assessed by plotting appropriate WHO Growth Charts. Child food poverty was measured using the UNICEF and WHO dietary diversity score.

Results: Of 110 participants, the adequacy of nutrition in energy and protein was found in 30 participants (27.27%) and 18 participants (16.36%), respectively. The proportion of moderate child food poverty was found to be 71 (64.54%). Unhealthy snacking patterns and poor feeding practices were present. Age, parents' education, socioeconomic status, type of family, and birth order of the child were found to be significantly associated with nutritional adequacy.

Conclusion: Children aged two to five years in the urban slum lacked adequate nutrition in energy and protein; most children were suffering from moderate child food poverty and had poor feeding practices in the urban slums.

## Introduction

Good health, nutrition, and well-being throughout the life cycle are fundamental prerequisites for a healthy and productive existence [1]. Crucially, each instance of feeding represents a critical opportunity to provide the essential energy, protein, vitamins, minerals, and micronutrients required for optimal child growth and development. The first five years are crucial for a child's growth, as overall physical and brain development occur during this time.

The right to nutrition is globally recognized as a fundamental right of every child [2]. But according to the UNICEF report, 47 million children under five worldwide are affected by wasting, 44 million by stunting, and 38.3 million by overweight. In India, the burden remains substantial, with 20.1 million children experiencing wasting, 40.3 million stunting, and 1.9 million overweight [3].

Malnutrition refers to an excessive or insufficient intake of nutrients, which can also result from an imbalance in essential nutrients or poor nutrient absorption [4]. One in three children has malnutrition, two in three children are not fed an adequate diet, and at least one in three children under five is either overweight or undernourished [5]. In India, this translates to approximately 53% of children are not growing well, i.e., they are either underweight, overweight, or stunted [6].

Diet is a crucial part of a child's growth. The Indian Council of Medical Research recommends an energy requirement of around 1,100-1,350 kcal/day and a protein requirement of 16-24 g/day for children under five [7]. But most children consume inadequate nutrition. Inadequate dietary intake in early childhood can lead to failure of children reaching their developmental potential. A poor diet can also increase their susceptibility to developing diet-related noncommunicable diseases like diabetes, chronic respiratory disorders, cardiovascular disease, and cancer [6].

To quantify the extent of dietary deprivation experienced by children, UNICEF has defined child food poverty as the inability of a child to consume a nutritionally adequate and diverse diet during early childhood. Child food poverty in the early childhood, i.e., in the first five years of life, can affect their physical growth, cognitive development, and their ability to achieve their full developmental potential. It is often observed that children suffering from child food poverty lead to poor school performance, which leads to lower income later in life. This creates a vicious cycle of poverty and inadequate nutrition.

Globally, one in four children (27%) is living in severe child food poverty in early childhood, amounting to 181 million children under five years of age. In India, over 70% of the children under five are living in child food poverty [2].

Many studies have focused on anthropometric measurements to assess malnutrition in preschool children. There is a knowledge gap regarding the current scenario of energy and protein adequacy in the diet and the proportion of child food poverty among preschool children in urban slum areas. Hence, this study is undertaken to assess the energy and protein adequacy in the diet and also to estimate the proportion of child food poverty in children aged two to five years in the urban slums. This study will provide valuable information on the nutritional status of children under five, informing targeted interventions.

Objectives

This study aimed to determine nutritional adequacy by assessing energy and protein adequacy, and to estimate child food poverty among children aged two to five years in the urban field practice area of Bangalore Medical College and Research Institute, Bengaluru, India.

## Materials and methods

Study design and setting

A community-based prospective observational study was conducted among children aged two to five years from October 2024 to December 2024 in the urban field practice area of Bangalore Medical College and Research Institute, Bengaluru, India. All children enrolled in Anganwadi aged two to five years residing in the area for a minimum of one year and mothers who were willing to provide informed consent were included. Those children who had acute illness at the time of study or those who were known cases of chronic diseases (like malabsorption syndrome, and respiratory and cardiac diseases) and differently abled children were excluded from the study.

Sample size and sampling technique

The sample size of 110 was calculated based on a previous study conducted by Kulsum et al., which reported that 22% of children aged 4-14 years had a diet adequate in protein and calories [8]. This calculation assumed that 18% of children aged two to five years have adequate nutrition with a 95% confidence level, 80% power, and 8% absolute precision. Children enrolled in Anganwadi were selected using simple random sampling, with a computerized random number generator.

Method of data collection

Within the urban field practice area, there were 10 Anganwadi centers. Details about the children registered at the 10 Anganwadi centers were collected. A list was made, including children aged two to five years, and numbered. Children were selected using a random number generator until the necessary sample size was attained. The selected children's addresses were collected from the Anganwadi, and their homes were personally visited; consent was obtained from the mothers. Data were collected using a pretested, validated, semistructured questionnaire. Mothers were educated about the spoons and cups used for serving the child at home to quantify the intake of food and beverages. The adequacy of energy and protein in the diet was assessed using the 24-hour recall method over a period of more than seven days. Dietary intake of the previous day was collected through telephone calls for the successive seven days. Mothers were advised not to alter the dietary pattern of their children during the study period. Mothers who did not attend the calls or those who did not own mobile phones were personally visited to collect the data. These measures helped to minimize both bias and attrition. Malnutrition was also assessed by measuring the weight, height, and mid-arm circumference of the child during the first visit. The collected data were kept confidential.

Assessment tool

The weight of the child was measured using a standardized weighing scale. The height of the child was measured using a stadiometer. The mid-upper arm circumference of the child was measured using a Shakir tape. Malnourishment was assessed by plotting the measured values in the appropriate WHO Growth Charts [9]. Child food poverty was measured using the WHO and UNICEF dietary diversity score.

Interpretation of the WHO and UNICEF Dietary Diversity Score

WHO and UNICEF dietary diversity score has eight defined food groups: 1) breast milk, 2) grains, white/pale starchy roots, tubers, and plantains, 3) beans, peas, lentils, nuts, and seeds, 4) dairy products (milk, infant formula, yogurt, and cheese), 5) flesh foods (meat, fish, poultry, and organ meats), 6) eggs, 7) vitamin A-rich fruits and vegetables, and 8) other fruits and vegetables.

If children are fed five or more groups per day, they are categorized as "no child food poverty," only three to four groups per day, they are categorized as "moderate child food poverty," and only zero to two groups per day, they are categorized as "severe child food poverty" [2].

Statistical methods

The data collected were entered into Microsoft Excel (Microsoft Corporation, Redmond, WA) and analyzed using IBM Statistical Package for the Social Sciences Statistics (version 25; IBM Corp., Armonk, NY). Descriptive statistics were represented as percentages, means, and standard deviations. The chi-square test was used to determine the association between the categorical variables. A p value of <0.05 was considered statistically significant.

## Results

Sociodemographic details about the participants

A total of 110 participants were included in the study. The majority of the children, 64 (58.18%), were aged between two and three years. The mean age of the children was 3.49 years (1.08 years). According to the Modified Kuppuswamy classification 2024 [10], the families predominantly belonged to the socioeconomic class of the upper and lower classes, 67 (60.91%). This was followed by children from the lower middle class, comprising 35 (31.81%) participants, and the lower class, with eight participants. Most of the participants' mothers were high school graduates, 65 (59.09%). Many of the study participants lived in nuclear families, 76 (69.09%).

Energy and protein adequacy in children

The energy and protein adequacy of children were assessed based on dietary consumption. The study participants were found to have only 30 (27.27%) and 18 (16.36%) of energy and protein adequacy, respectively. The association of various factors with energy and protein adequacy is given in Table 1.

**Table 1:** Association of various factors with energy and protein adequacy Cross-tabulations were performed to examine the association between various categorical variables and energy/protein adequacy. Statistical significance was assessed using the chi-square test ^*^p < 0.05 is considered statistically significant

Factors	Energy adequacy	Energy inadequacy	ꭓ² value	p value	Protein adequacy	Protein inadequacy	ꭓ² value	p value
Age (in years)								
2-3	22 (34.37%)	42 (65.62%)	3.892	0.049^*^	0 (0%)	64 (100%)	29.943	0.001^*^
4-5	8 (17.39%)	38 (82.61%)			18 (39.13%)	28 (60.87%)		
Age of mothers (in years)								
20-29	18 (27.69%)	47 (72.31%)	0.014	0.905	14 (21.54%)	51 (78.46%)	3.109	0.078
30-40	12 (26.66%)	33 (73.34%)			4 (8.89%)	41 (91.11%)		
Education level of mother								
Illiterate	4 (33.33%)	8 (66.67%)	9.735	0.045^*^	4 (33.33%)	8 (66.67%)	8.071	0.089
Primary school	4 (33.33%)	8 (66.67%)			0 (0%)	12 (100%)		
Middle school	0 (0%)	17 (100%)			5 (29.41%)	12 (70.59%)		
High school	22 (33.84%)	43 (66.16%)			9 (13.85%)	56 (86.15%)		
Graduate	0 (0%)	4 (100%)			18 (16.36%)	92 (83.64%)		
Type of family								
Nuclear	20 (26.32%)	56 (73.68%)	13.294	0.001^*^	14 (18.42%)	62 (81.58%)	3.817	0.148
Three generation	10 (55.56%)	8 (44.44%)			4 (22.22%)	14 (77.78%)		
Joint	0 (0%)	16 (100%)			0 (0%)	16 (100%)		
Socioeconomic class								
Lower	0 (0%)	8 (100%)	26.474	0.001^*^	0 (0%)	8 (100%)	4.222	0.121
Lower middle	0 (0%)	35 (100%)			9 (25.71%)	26 (74.29%)		
Upper lower	30 (44.78%)	37 (55.22%)			9 (13.43%)	58 (86.57%)		
Birth order of child								
1	18 (38.29%)	29 (61.71%)	9.639	0.022	5 (10.64%)	42 (89.36%)	27.238	0.001^*^
2	12 (26.67%)	33 (73.33%)			4 (88.89%)	41 (91.11%)		
3	0 (0%)	14 (100%)			9 (64.29%)	5 (35.71%)		
4	0 (0%)	4 (100%)			0 (0%)	4 (100%)		
Inference from weight for height plotted in growth charts								
Moderate acute malnourished	1 (25%)	3 (75%)	0.011	0.917	1 (25%)	3 (75%)	0.226	0.634
Normal	29 (27.36%)	77 (72.64%)			17 (16.04%)	89 (83.96%)		
Inference from mid upper arm circumference								
Severe acute malnourished	0 (0%)	1 (100%)	0.392	0.822	1 (100%)	0 (0%)	5.896	0.052
Moderate acute malnourished	01 (25%)	3 (75%)			0 (0%)	4 (100%)		
No malnutrition	29 (27.62%)	76 (72.38%)			17 (16.19%)	88 (83.81%)		
Child food poverty index								
Severe child food poverty	0 (0%)	4 (100%)	15.647	0.001^*^	0 (0%)	4 (100%)	1.989	0.370
Moderate child food poverty	12 (16.90%)	59 (83.10%)			14 (19.72%)	57 (80.28%)		
No child food poverty	18 (51.43%)	17 (48.57%)			4 (11.43%)	31 (88.57%)		

The diet adequacy and various factors have been shown in Table 2.

**Table 2:** Association between adequacy pattern and various factors Nutritional adequacy was classified into four categories. Cross-tabulations were performed using the chi-square test to assess associations between various factors and nutritional adequacy status P-C-: inadequacy in both protein and energy; P-C+: inadequate in protein and adequate in energy; P+C-: adequate in protein and inadequate in energy; P+C+: adequate in both energy and protein ^*^p value <0.05 is statistically significant

Factors	No.	Adequacy pattern				ꭓ²	p value
		P-C-	P-C+	P+C-	P+C+		
Age groups (in years)							
2-3	64	42 (65.63%)	22 (34.37%)	0 (0%)	0 (0%)	33.317	0.001*
4-5	46	24 (52.17%)	4 (8.69%)	4 (30.45%)	04 (8.69%)		
Socioeconomic status							
Lower	8	08 (100%)	0 (0%)	0 (0%)	0 (0%)	31.366	0.001^*^
Lower middle	35	26 (74.28%)	0 (0%)	9 (25.72%)	0 (0%)		
Upper lower	67	32 (47.76%)	26 (38.81%)	5 (7.46%)	4 (5.97%)		
Did your child have any recent episode of acute diarrheal disease in the last one month?							
Yes	8	8 (100%)	0 (0%)	0 (0%)	0 (0%)	5.752	0.109
No	102	58 (56.86%)	26 (25.29%)	14 (13.73%)	4 (3.92%)		
Did your child have any recent episodes of acute respiratory diseases in the last one month?							
Yes	31	16 (51.61%)	10 (32.26%)	5 (16.13%)	0 (0%)	3.826	0.281
No	79	50 (63.29%)	16 (20.26%)	9 (11.39%)	4 (5.06%)		
When was complementary feeding started?							
<6 months	26	13 (50%)	08 (30.77%)	5 (19.23%)	0 (0%)	20.699	0.002^*^
At 6 months	42	32 (76.19%)	06 (14.29%)	0 (0%)	4 (9.52%)		
>6 months	42	21 (50%)	12 (28.57%)	9 (21.43%)	0 (0%)		
Child food poverty index							
No child food poverty	35	17 (48.57%)	14 (40%)	0 (0%)	4 (11.43%)	24.498	0.001^*^
Moderate child food poverty	71	45 (63.38%)	12 (16.90%)	14 (19.72%)	0 (0%)		
Severe child food poverty	4	4 (100%)	0 (0%)	0 (0%)	0 (0%)		

Child food poverty index in children

**Figure 1:**
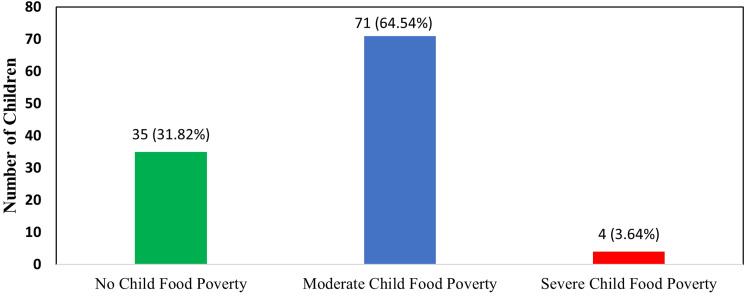
Child food poverty index Child food poverty was classified according to WHO and UNICEF dietary diversity guidelines based on consumption from eight defined food groups. Children consuming five or more food groups in the previous day were categorized as having no child food poverty; those consuming three to four groups as moderate child food poverty; and those consuming zero to two groups as severe child food poverty

The majority of children, 71 (64.54%), were categorized as moderate child food poverty. A significant proportion, 35 (31.81%), exhibited no child food poverty. Conversely, severe child food poverty was identified in a smaller subset of four children (3.63%). These findings are represented in Figure 1.

**Figure 2:**
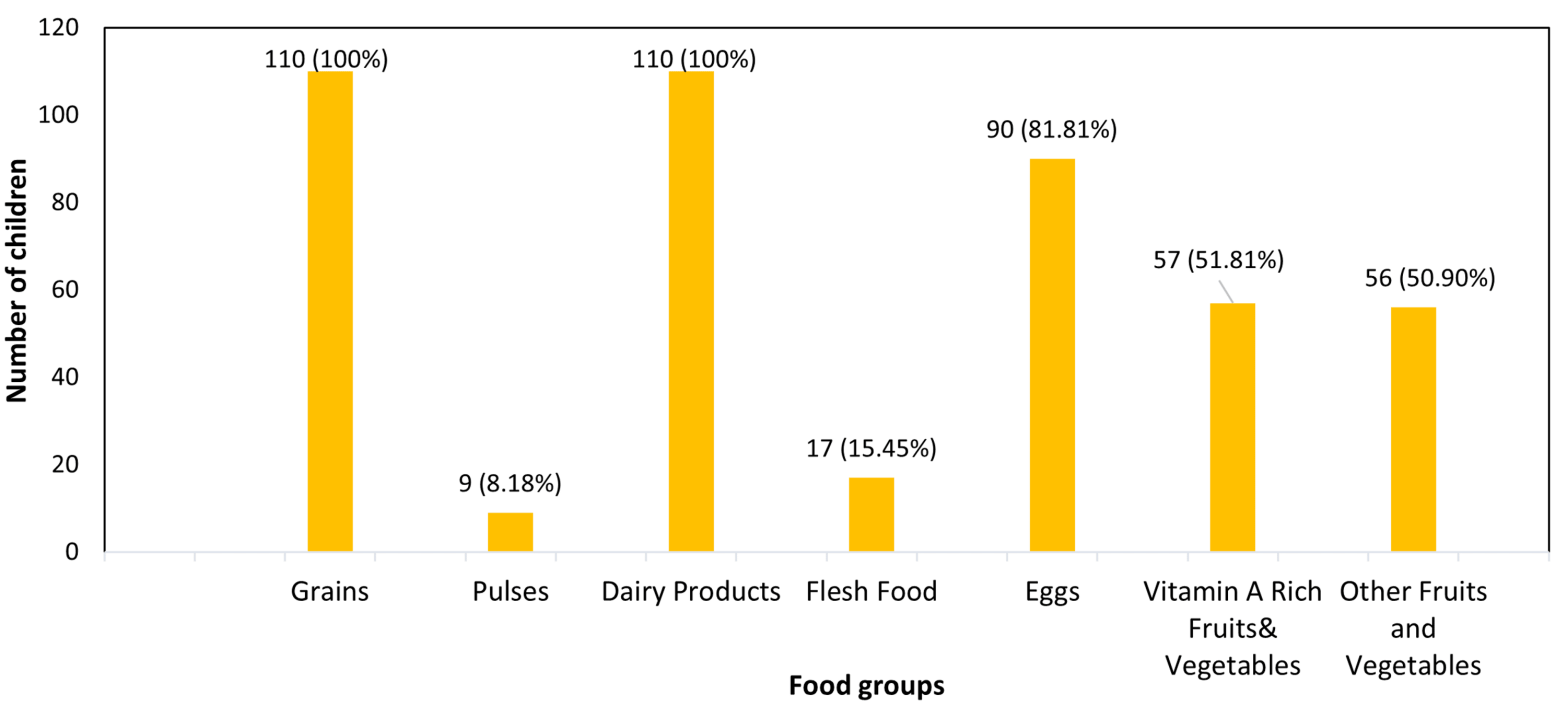
Consumption pattern of food groups from 24-hour recall Food group consumption among children as reported in a 24-hour dietary recall, based on the WHO and UNICEF dietary score. The figure shows the number and percentage of children who consumed each group Flesh foods include meat, fish, poultry, and organ meats Other fruits and vegetables include fruits and vegetables that are not rich in Vitamin A

The consumption patterns of diverse food groups by children over a 24-hour period are illustrated in Figure 2.

Association between energy and protein adequacy and child food poverty

The majority of the children suffering from moderate child food poverty, 81 (73.63%), also suffered from energy inadequacy, 59 (53.63%), and protein inadequacy, 57 (51.81%). Child food poverty was significantly associated with energy adequacy (p = 0.001) and not significantly associated with protein adequacy (p = 0.370).

Food taboos and food practices among parents

During the period when the child was sick, a small percentage of families, four families (3.63%), prohibited nonvegetarian spicy food, ice cream, and chocolate. A more significant portion, 25 (22.72%), opted for an exclusive liquid diet of diluted milk and ganji, while 13 families (11.81%) chose a bland diet of idly and dosas. Additionally, four families (3.63%) restricted foods such as jackfruit, curd, pineapple, and sapota based on their beliefs that these foods cause "cold" or "heat." The morning and evening snacks of children are given in Table 3.

**Table 3:** Different snacks consumed by children in the urban slums

Snacks	Morning snacks	Evening snacks
Packaged potato chips	44 (40%)	15 (13.63%)
Biscuits	14 (12.72%)	30 (27.27%)
Homemade fried chips	20 (18.18%)	22 (20%)
Fruit	12 (10.91%)	8 (7.27%)
Milk	4 (3.63%)	18 (16.36%)
Chocolate	9 (8.18%)	6 (5.45%)
Packaged instant noodles	7 (6.36%)	11 (10%)

## Discussion

The study assessed the energy and protein adequacy of children under five through 24-hour recalls over seven days and estimated child food poverty among the participants. In the present study, the majority of the population belongs to the upper and lower classes. Most of the mothers have passed high school. The study revealed that only 30 (27.27%) of the children had an adequate energy intake, and only 18 (16.36%) met the protein adequacy standard. This indicates a high prevalence of energy inadequacy, 80 (72.72%), and protein inadequacy, 92 (83.6%), among the participants.​ In our study, the number of children with a diet adequate in both protein and energy (P+C+) is four (3.63%), whereas children who have an inadequate energy and nutrition (P-C-) are 66 (60%). This contrasts with the findings of Kulsum et al., who conducted a study in the urban slums of Mysore. They reported that 27% of children aged four to six years showed inadequacy in both energy and protein (P-C-), with no children in that age group having adequate levels of both energy and protein (P+C+) [8]. This difference in the inadequacy of energy and protein could be attributed to the time gap between the studies, which is due to the age group. In a study conducted by Anjali et al. among adolescent girls, energy adequacy was observed in 44 participants (11.4%), while protein adequacy was observed in 78 (18.5%). These findings indicate the need for targeted research across different population subgroups and the development of age-specific nutritional interventions [11]. The present study reflects the contemporary dietary landscape, characterized by the easy availability of ultraprocessed foods and a decline in the consumption of traditional protein sources, such as pulses and meat products, which have contributed to higher rates of nutritional inadequacy.

The maternal education has shown a significant association with energy adequacy. The type of family also showed a significant association. But the prevalence of nuclear families was higher in the urban areas, which can affect the extended family support, which otherwise would have helped the working parents in caregiving for their children.

In the present study, the Child Food Poverty Index indicated that 71 (64.54%) of children lived in moderate food poverty, 35 (31.81%) had no food poverty, and four (3.63%) experienced severe food poverty. Diets predominantly included grains, dairy products, and eggs, with limited consumption of flesh foods and pulses. ​These findings are consistent with global reports. UNICEF's 2023 report highlighted that in India, 40% of children under five suffer from severe food poverty, and 36% suffer from moderate child food poverty [2]. While our study's figures show a lower prevalence of severe food poverty, four (3.63%), the percentage of children in moderate food poverty aligns with the national statistics. The discrepancy in severe food poverty could be due to regional variations and the specific demographic of our study population. In a study conducted by Singh et al. in Bihar, the 24-hour dietary recall revealed consumption of starchy staples by 193 (95.5%), legumes, nuts, and seeds by 150 (74.3%), eggs by 53 (26.2%), and milk and milk products by 173 (85.6%). In contrast, our findings revealed that 90 participants (81.81%) consumed eggs, and all 110 participants (100%) consumed milk and milk products, whereas only nine participants (8.18%) reported consuming pulses, highlighting notable differences in dietary patterns between the two study populations. The variation between Bihar and Karnataka in dietary diversity may be due to cultural preferences, with Bihar favoring plant-based proteins like pulses and Karnataka showing higher intake of eggs and milk, likely influenced by state nutrition programs such as Ksheera Bhagya. Socioeconomic differences, agricultural availability, and government feeding schemes further contribute, as Karnataka's institutional support ensures access to animal-based foods, while Bihar relies more on affordable legumes [12].

The majority of the children consumed a diet from food groups such as grains, dairy products, and eggs. Consumption of flesh food like chicken and fish is only restricted to once or twice a week. Only a few children consume pulses like beans, peas, lentils, and nuts. This study revealed a common practice among families of restricting sick children to a limited diet of two or fewer food groups, often consisting of rava ganji, idli, or dosa alongside diluted milk. This practice of relying on a very limited range of food groups during illness raises concerns about adequate nutrient intake and its potential to compromise the child's overall recovery. A study by Benakappa and Shivamurthy on children's dietary habits found similar beliefs across different health conditions. In children aged two to five years, 72% were not given nonvegetarian food. Among children with respiratory illnesses (n = 53), the most preferred foods were idli (24.61%), rice (18.46%), and bread (16.98%). For children with gastroenteritis and dysentery (n = 14), the favored solid foods were rice (28.57%) and idlis (21.42%), while the preferred liquids were milk (85.7%) and buttermilk (21.42%). The study also noted that meat and spicy foods were commonly avoided by these children [13].

The study noted that children's morning and evening snacks mainly consisted of ultraprocessed foods like biscuits, potato chips, and packaged instant noodles. Such foods are often high in calories but low in essential nutrients, which can lead to insufficient micronutrient intake and potentially diminish their appetite for more nutritious food, increasing their inclination toward less healthy options. Athavale et al. found that 50% of children in low-income communities consume junk food daily, with such options readily available within a five-minute walk from their homes [14]. A study conducted by Agarwal et al. in Gujarat reported findings consistent with the present study. It was observed that 2,836 (30.8%) children consumed one or more packets of junk food per day, and 1,584 (17.3%) children frequently consumed packaged juices. These patterns reflect the increasing trend of unhealthy dietary practices among children, underscoring the need for targeted nutritional interventions and awareness programs [15].

The study has a few limitations to consider. First, the study collected a 24-hour recall diet for seven days. There are chances of bias, such as forgetting to mention some of the food items eaten by the child, or thinking the quantity of some food items consumed is too small to be of importance to be mentioned, or bias in the quantity of food mentioned. Second, the study comprises only children aged two to five years from the slums of the urban field practice area of Bangalore Medical College and Research Institute. Employing a multicentric approach involving other urban slums and rural areas could have improved generalizability to comment on the adequacy of nutrition and child food poverty in our country. Further research can also include assessing micronutrient deficiency in the children.

The study highlights the need to implement targeted awareness programs for mothers on preparing nutrient-dense dietary recipes, along with providing appropriate training for Accredited Social Health Activist and Anganwadi workers on Maternal, Infant, and Young Child Nutrition. Mothers should be encouraged to provide diverse food groups to the child. Snacks like chikki, fruits, egg, and milk should be encouraged. Training mothers to prepare nutrient-dense foods is essential. This includes practices such as the step-by-step introduction of ragi cereal after six months of age, the gradual inclusion of fruits as the last food group, and encouraging children to start consuming household foods by one year of age. Additionally, actively encouraging mothers to collect their entitled food rations from Anganwadi centers on a regular basis can further enhance access to nutritious food options for these vulnerable children.

## Conclusions

The study brought light to the concerning situation, where most of the children are consuming an inadequate diet in energy and protein. Consequently, most children are experiencing Moderate Child Food Poverty. Factors such as the high prevalence of nuclear families, socioeconomic status, improper initiation of complementary feeding, the consumption of ultraprocessed foods, and the limited intake of diverse food groups, particularly flesh foods and pulses, contribute significantly to this nutritional challenge.

## References

[1] (2024). Poshan power note strengthening the evidence base through the comprehensive national survey. prevalence and burden of malnutrition in children under five years old in India, NAFS-5I, (2019-21). https://www.unicef.org/india/reports/poshan-power-note-strengthening-evidence-base-through-comprehensive-national-survey.

[2] (2024). Child food poverty. Nutrition deprivation in early childhood. Child nutrition report. https://data.unicef.org/resources/child-food-poverty-report-2024/.

[3] (2025). The State of Food Security and Nutrition in the World 2025 - addressing high food price inflation for food security and nutrition. https://doi.org/10.4060/cd6008en.

[4] (2024). Malnutrition. Factsheet. https://www.who.int/news-room/fact-sheets/detail/malnutrition.

[5] (2024). Nutrition, for every child. Improving young children's diet during the complementary feeding time. UNICEF Nutrition-Strategy.

[6] (2024). Asia and the Pacific regional overview of food security and nutrition 2020: maternal and child diets at the heart of improving nutrition. https://doi.org/10.4060/cb2895en.

[7] Indian Council of Medical Research (2025). A brief note on nutrient requirements for Indians, the recommended dietary allowances (RDA) and the estimated average requirements (EAR), ICMR - NIN. NIN.

[8] Kulsum A Jr, Lakshmi JA, Prakash J (2008). Food intake and energy protein adequacy of children from an urban slum in Mysore, India - a qualitative analysis. Malays J Nutr.

[9] (2025). Child growth standards. https://www.who.int/tools/child-growth-standards/standards.

[10] Javalkar SR, Shalini H, Davalagi SB, Vidya GS (2024). Socio economic status assessment in India: history and updates for 2024. Int J Community Med Public Health.

[11] Anjali G, Judith NA, Shobha Shobha, Meenakshi G (2018). Dietary intake of macronutrients and micronutrients among adolescent girls: a cross sectional study. Clin Epidemiol Glob Health.

[12] Singh T, Kumar S, Sinha S, Kumar S (2025). Dietary diversity score and its sociodemographic determinants in school-aged children: cross-sectional baseline findings from a quasi-experimental study. Cureus.

[13] Benakappa AD, Shivamurthy P (2012). Beliefs regarding diet during childhood illness. Indian J Community Med.

[14] Athavale P, Khadka N, Roy S, Mukherjee P, Chandra Mohan D, Turton BB, Sokal-Gutierrez K (2020). Early childhood junk food consumption, severe dental caries, and undernutrition: a mixed-methods study from Mumbai, India. Int J Environ Res Public Health.

[15] Agarwal N, Choraria N, Howale SD (2025). Prevalence of packaged snacks, beverages, and junk food consumption among school-going children and parental perceptions: a cross-sectional study. Int J Sci Res.

